# More than just emotional intelligence online: introducing “digital emotional intelligence”

**DOI:** 10.3389/fpsyg.2023.1154355

**Published:** 2023-05-02

**Authors:** Catherine Audrin, Bertrand Audrin

**Affiliations:** ^1^University of Teacher Education (Vaud), Lausanne, Switzerland; ^2^EHL Hospitality Business School, HES-SO, University of Applied Sciences and Arts Western Switzerland, Delémont, Switzerland

**Keywords:** emotional intelligence, digital competence, digital skills, digital emotional intelligence, ability emotional intelligence, trait emotional intelligence

## Abstract

The ubiquitous nature of emotional intelligence, as a central theme in every aspect of our lives—be it at work, school, or home—coupled with the growing prevalence of digital interactions, makes it fundamental to develop our understanding of emotional intelligence in a digital world. However, the digital world represents more than just a contextual factor to consider, as interactions in digital environments require digital competence. The objective of this paper is to conceptualize “digital emotional intelligence” as the integration of both emotional intelligence and digital competence. The model we propose posits that trait emotional intelligence predicts attitudes toward digital competence, while digital ability emotional intelligence is predicted by digital competence skills and digital competence knowledge. Using a self-reported questionnaire on 503 respondents, a structural equation model highlights a positive relationship between trait emotional intelligence and attitudes toward digital competence.

## 1. Introduction

Emotional intelligence (EI) plays a central role in our lives and is related to multiple positive outcomes. Since its coining by Salovey and Mayer (1990) and its popularization by Goleman (1995), EI has gained widespread interest in academic circles, be it in education, organizations, or society in general. In education research, results have shown the importance of EI in academic contexts (Garner, 2010). In organization studies, research has demonstrated its impact on job satisfaction (Miao et al., 2017a) and organizational citizenship behavior (Miao et al., 2017b). EI has also been found to have a negative influence on burnout (Szczygiel and Mikolajczak, 2018) and counterproductive work behavior (Miao et al., 2017b).

However, the current understanding of EI does not account for the digital context in which we all interact, work, and live today. One notable exception comes from scholars in the emotion regulation literature who have called for research on “digital emotion regulation” (Wadley et al., 2020; Smith et al., 2022). Wadley and colleagues define it as a “process in which people evaluate their emotions in relation to their current goals and decide whether to modify them and, if so, select which regulation strategy to use” (Wadley et al., 2020; p. 413) and investigate (1) how digital technology may be used to regulate emotions, and (2) how digital technologies may impact people's emotions. This emphasizes the relevance of the digital world and its importance in shaping the way we experience emotions.

Over the last 20 years, digital competence (DC) (also referred to as digital literacy or digital skills) has gained interest from scholars and policymakers as a multifaceted competence that needs to be developed in learners (Zhao et al., 2021), workers (Oberländer et al., 2020), and citizens (Vuorikari et al., 2022). Some aspects of DC, such as technical skills, represent strict entry barriers to the digital world, while others, such as digital communication skills, drastically shape our interactions and emotional experiences online (Sánchez-Caballé et al., 2020). Thus, it seems very difficult to fully experience emotions in the digital world without DC. Conversely, EI can have a strong impact on DC, especially in terms of adopting appropriate behaviors online and addressing ethical issues.

In that respect, the relationship between DC and EI might be reciprocal. Our purpose is thus to question the integration of DC and EI in a digital environment. To achieve this, this paper reviews literature on EI and DC to map out the variety of approaches to both concepts and their implications when it comes to modeling the relationship between them. Specifically, we discuss the specificities of both trait and ability approaches to EI and their relationship to DC. We also present the various frameworks of DC and their similarities and differences as well as their overall purpose, to discuss what DC ultimately consists of. Drawing on the literature review, we develop a conceptual model for “digital emotional intelligence” (dEI), comprising two key conceptual linkages between trait emotional intelligence (TEI) and DC, and between DC and ability emotional intelligence (AEI). More specifically, our conceptual model of dEI posits that TEI will predict DC-Attitudes, while digital AEI (dAEI) is predicted by DC-Skills and DC-Knowledge. Based on these propositions, we formulate a hypothesis centered on the relationship between TEI and DC-Attitudes that we empirically test.

### 1.1. Emotional intelligence

EI was introduced by Salovey and Mayer (1990; p. 189) as “the subset of social intelligence that involves the ability to monitor one's own and others' feelings and emotions, to discriminate among them and to use this information to guide one's thinking and actions.” Since this seminal work, many conceptualizations and definitions have emerged around it. In the following, we will focus on TEI and AEI.

### 1.2. Trait emotional intelligence

One type of model describes EI as a dispositional trait (Vesely Maillefer et al., 2018). This approach grounds EI in a personality perspective and differential psychology (Petrides et al., 2016). In this conceptualization, TEI refers to individuals' emotional dispositions and focuses on how people perceive their emotional world (Petrides et al., 2016). TEI is defined as “a constellation of emotional self-perceptions” (Petrides, 2010; p. 137) such as adaptability, empathy, emotion expression and perception and self-esteem, among others. TEI can thus be assessed using self-reported questionnaires and has also been called “trait emotional self-efficacy” (Petrides et al., 2016; p. 339).

### 1.3. Ability emotional intelligence

A second type of model defines EI as an ability. AEI is “the ability to reason validly with emotions and with emotion related information, and to use emotions to enhance thought” (Mayer et al., 2016; p. 296). Based on this definition, AEI is usually measured through performance in tasks (Olderbak et al., 2019).

The most prominent model focusing on AEI is the model of Salovey and Mayer (Salovey and Mayer, 1990; Mayer and Salovey, 1997). Their model suggests that AEI comprises four factors: emotion perception, facilitation of thought using emotions, emotion understanding, and emotion management. Recent studies have suggested that a model containing three factors (removing the “using emotions” factor) presents a better empirical fit (Vesely Maillefer et al., 2018). Building on that, we will rely on this three-factor model in the rest of the paper.

Emotion perception refers to one's capacity to identify emotions accurately. This factor may be oriented toward oneself (i.e., the ability to pay attention to one's own physical and psychological state regarding emotions) or toward others (i.e., the tendency to be sensitive to others' emotions)—(Mayer et al., 1999). This factor also refers to the capacity to identify emotional content in its environment, and notably to assess if it is accurate or not (Mayer et al., 2016). Understanding emotions—the second factor of the model—refers to the ability to understand that emotions can be connected to each other and that they can change across situations and time (Rivers et al., 2007). This also refers to knowing which situations can lead to certain emotions (Mayer et al., 2016). The third factor of the model (managing emotions) refers to the ability of people to regulate their emotions as well as others' emotions. This factor thus taps into to the capacity to manage emotions to achieve a desired outcome, to assess different strategies that can be used to control the emotion that is being felt and to choose to engage or disengage with the emotion felt, depending on one's need.

### 1.4. Digital competence

To be active in a digital context, digital competence (DC) is required. DC has been shown to be not only a right but also a requirement of citizens, as it is necessary to be functional now (Ferrari, 2012). Research has revealed a strong interest in the study of DC in (tertiary) educational settings (Zhao et al., 2021; Audrin and Audrin, 2022). Zhao et al. (2021) highlight that different theoretical frameworks regarding digital competence co-exist. Audrin and Audrin (2022) further reveal that the field suffers from a lack of clarity regarding the terminology (i.e., digital literacy, digital skills, digital competence, 21st century skills, …). Despite this lack of agreement, literature agrees that DC is not only constituted of technological skills, but that it encompasses multiple literacies (Sánchez-Caballé et al., 2020). Moreover, as highlighted by Sillat et al. (2021), digital competence does not only refer to skills, but it refers to a wider sense of competence, as it comprises knowledge, skills and attitudes.

Three DC frameworks have gained traction in the literature over the last few years, coming from 91) the UK Department for Education, (2) the European Commission's science and knowledge service, and (3) van Laar et al. (2018). These frameworks have different purposes and are aimed at different groups of actors. While the UK Department for Education's framework is intended for anyone who wants to improve their digital skills (Department for Education., 2019), the DigComp framework aims to “create an agreed vision of what is needed in terms of competence to overcome the challenges that arise from digitization in almost all aspects of modern lives” (Vuorikari et al., 2022; p. (4). In contrast, the framework developed by van Laar et al. (2018) aims at developing “a set of reliable measures that focus on the frequency of activities that working professionals perform to assess each core 21st-century digital skill” (van Laar et al., 2018; p. 2185).

While the frameworks differ in how they are structured and operationalized, several main dimensions appear as the core of DC: Information (using digital technology to search, filter, organize information and digital content), communication and collaboration (using digital technology to transmit and share information, but also to interact with others), critical thinking, problem-solving & decision-making (using digital technology to make informed judgments, assessing the information available online, and sorting through relevant data online), safety and legality (adopting security measures, but also behaving in a respectful way online), digital foundation skills (having the basic technical skills to use digital technologies). Making sense of these categories is helpful as it makes it possible to identify what DC is about. However, this only helps scrap the surface of DC as each of these dimensions entails a wide variety of sub-dimensions.

Another way to look at these frameworks is to focus on the components that constitute their sub-dimensions, namely knowledge, skills, and attitudes (Hämäläinen et al., 2021; Vuorikari et al., 2022), relying on the conception of competence as the integration of knowledge, skills and attitudes (Baartman and De Bruijn, 2011; Lizzio and Wilson, 2004; Seufert et al., 2021). Vuorikari et al. (2022) further develop this distinction in their framework. In their sense, knowledge is “the outcome of the assimilation of information through learning” (Vuorikari et al., 2022; p. 3). Knowledge thus bears a very informational dimension: in the context of DC, it is the knowledge that individuals have about the digital world, its tools, its rules, and how to behave online. In contrast, skills can be defined as “the ability to apply knowledge and use know-how to complete tasks and solve problems” (Vuorikari et al., 2022; p. 3). Skills thus refer to “doing or acting in practice, involving motor skills as well as cognitive skills” (Baartman and De Bruijn, 2011; p. 127). As such, they are embedded in practice: in the context of DC, it is the ability of individuals to carry out tasks in the digital world. Finally, attitudes is tied with individuals' beliefs (Hämäläinen et al., 2021), and can be defined as “an individual's predisposition to respond favorably or unfavorably to an object, person, or event” (Aslan and Zhu, 2017; p. 555). Attitudes can thus be considered as more of predispositions toward action: in the context of DC, it is the tendency that individuals will have to behave in a certain way in the digital world. Distinguishing between knowledge, skills, and attitudes helps understand the variety of dimensions of competence required to be digitally competent.

In summary, the literature on DC is still in the process of standardizing its definition and assessment. This overview shows that some dimensions emerge as important in DC, such as: information, communication and collaboration, critical thinking, problem-solving & decision-making, safety and legality, digital foundation skills. On top of these dimensions, a transversal approach to DC focuses on the variety of its components and identifies different sets of knowledge, skills, and attitudes within each dimension.

## 2. An integrated model of digital emotional intelligence

**Figure 1:**
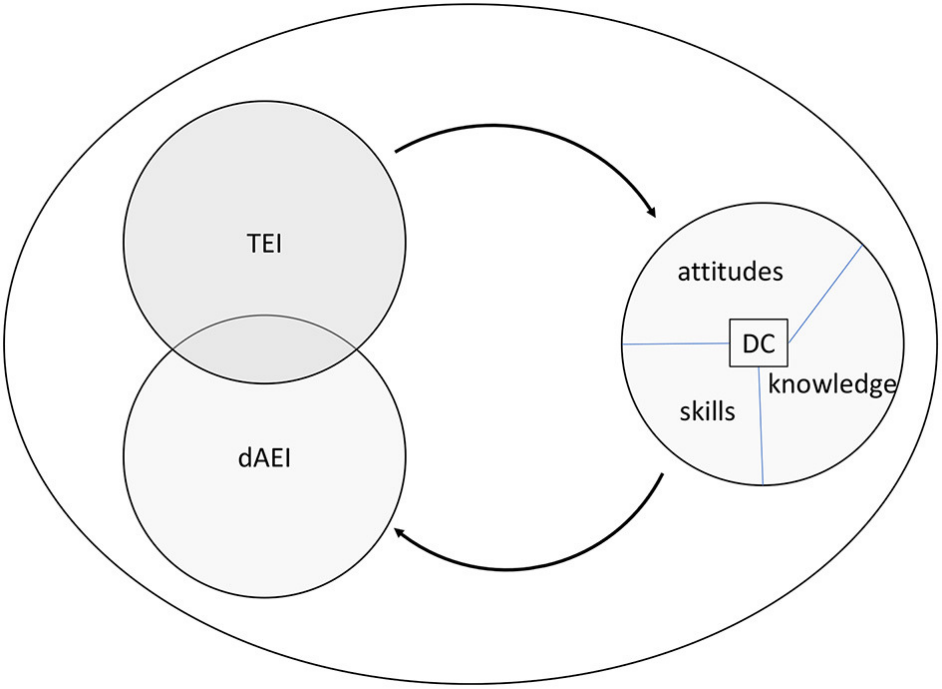
Proposed conceptual model of dEI.

The objective of this paper is to model the relationship between DC and EI in a digital environment, providing a conceptualization of “digital emotional intelligence” (dEI) that goes beyond solely “EI in a digital world” but rather as deeply integrated with DC. Based on our literature review of the concepts, considering both TEI and AEI as well as the diverse knowledge, skills, and attitudes that compose DC, we represent the conceptual model depicted in Figure 1. To build this model, we followed the steps recommended by Thatcher and Fisher (2022).

This conceptual model of dEI posits two relationships: (1) TEI and DC-Attitudes, (2) dAEI and DC-Knowledge and Skills. Note that we hereafter refer to digital AEI (dAEI) instead of simply AEI, as we formulate the proposition that DC-Knowledge and DC-Skills may allow and enhance a new form of AEI that is specific to the digital world. Our propositions are the following:

(1) TEI will predict DC-Attitudes
(2) DC-Knowledge will predict dAEI
(3) DC-Skills will predict dAEI.

In the following sections, we will justify, detail, and illustrate each of these relationships with examples. In the last part of the paper, we conduct an empirical test to investigate the proposed relationship between TEI and DC-Attitudes.

### 2.1. TEI and DC-attitudes

In our model of dEI, we formulate the proposition that TEI will predict DC-Attitudes. This assumption is based on the approach of TEI as a disposition toward action (Vesely Maillefer et al., 2018) which impacts attitudes (O'Connor et al., 2019). Studies using TEI have linked it with work attitudes, such as job satisfaction and organizational citizenship behavior (Miao et al., 2017a). As highlighted earlier, attitudes are conceptualized as “an individual's predisposition to respond favorably or unfavorably to an object, person, or event” (Aslan and Zhu, 2017; p. 555). In the context of DC, attitudes can be considered as predispositions toward action, i.e., the tendency that individuals will have to behave in a certain way in the digital world. Therefore, it makes sense to conceptualize TEI as an antecedent of individuals' DC-Attitudes. An analysis of the examples of attitudes presented in DigComp 2.2 (Vuorikari et al., 2022) allows to clearly identify the link with TEI. The dimensions of TEI—wellbeing, self-control, emotionality, and sociability—(Petrides et al., 2016) are apparent throughout the examples of key attitudes for DC. In the following, we are going to provide illustrations of some selected items from DC-Attitudes and how they display a specific form of TEI.

Several items of DC-Attitudes (“being inclined to focus on positive impacts and avoiding the negative impacts of digital media, such as overuse, addiction, and compulsive behavior”; “being open to explore alternative pathways to find solutions to produce digital content”; Vuorikari et al., 2022) can be tied with the wellbeing dimension of TEI. The wellbeing dimension of TEI comprises optimism, self-esteem beliefs and trait happiness (Petrides et al., 2016) can be identified in the selected items that present optimism (e.g., “focus on positive impacts”) and self-esteem (e.g., “being open to find solutions”) as part of DC attitudes.

Several items of DC-Attitudes (“intentionally avoiding distractions and aiming to avoid information overload when accessing and navigating information, data and content”; “being motivated to co-design and co-create new products and services using digital devices to create economic or social value for others”; Vuorikari et al., 2022) can be tied with the self-control dimension of TEI. The self-control dimension of TEI notably encompasses impulsiveness and stress management (Petrides et al., 2016) can be identified in the selected items that show an attitude toward impulse control (e.g., “intentionally avoiding distractions”), and stress management (e.g., “aiming to avoid information overload”).

Several items of DC-Attitudes (“being inclined to help others to improve their digital content”; “willing to adapt an appropriate communication strategy depending on the situation and digital tool: verbal strategies, non-verbal strategies, visuals strategies or mixed strategies”; Vuorikari et al., 2022) can be tied with the emotionality dimension of TEI. The emotionality dimension of TEI refers to emotional expression, empathy, emotion perception and relationships' quality (Petrides et al., 2016) and can be identified in the selected items that show an attitude toward emotion perception and expression (e.g., “willing to adapt appropriate communication strategy”) and relationships (e.g., “being inclined to help others…”).

Several items of DC-Attitudes (“being concerned that much online information and content may not be accessible to people with a disability, for example to users who rely on screen reader technologies to read aloud the content of a web page”; “considering ethics as one of the core pillars when developing or deploying AI systems”; “encouraging everyone to express their own opinions constructively when collaborating in digital environments, willing to help others to improve their digital competencies, building on their strengths and mitigating their weaknesses”; Vuorikari et al., 2022) can be tied with the sociability dimension of TEI. The sociability dimension of TEI notably focuses on assertiveness and awareness (Petrides et al., 2016) and can be identified in the selected items that show an attitude toward others (e.g., “willing to help others,” “encouraging everyone to express their own opinions”) in the digital context. These examples provide an illustration of the way in which the dimensions of TEI could be reflected in DC-Attitudes. Altogether, this exercise shows how predispositions related to emotional intelligence can influence and shape DC-Attitudes.

### 2.2. dAEI and DC-knowledge and-skills

In our model of dEI, we formulate the proposition that dAEI (digital-AEI) will be predicted by DC-Knowledge and DC-Skills. This assumption is based on the approach of AEI that conceptualizes it as an ability (Mayer et al., 2016) that can be developed (Zeidner et al., 2002). Literature further highlights that training may be particularly efficient when they include instructions (designed to enhance *knowledge*) and practice with feedback (designed to enhance *skills*) (Blanch-Hartigan et al., 2016). The same reasoning can be applied to the digital context, in which DC-Knowledge and DC-Skills should participate in improving individuals' dAEI. Knowledge is mostly informational (Baartman and De Bruijn, 2011) and, in the context of DC, it refers to what individuals know about the digital world, its tools, its rules, and how to behave online. Skills are embedded in practice (Baartman and De Bruijn, 2011) and, in the context of DC, it is the ability of individuals to carry out tasks in the digital world. In our model, we suggest that both knowledge of the digital world and skills to carry out tasks in the digital world are likely to impact the ability of individuals to reason validly with emotions and emotions-related information in a digital context (dAEI). Thus, we conceptualize DC-Knowledge and DC-Skills as antecedents of dAEI. An illustration of examples of knowledge and skills presented in DigComp 2.2 (Vuorikari et al., 2022) allows to identify the link with AEI very clearly. In the following, we are going to provide illustrations of some selected items of (1) DC-Knowledge and (2) DC-Skills taken from Vuorikari et al. (2022) and discuss how these can impact dAEI. The following examples only represent a selection of DC-Knowledge and DC-Skills that impact dAEI and have been chosen based on their relevance. Many other examples could have been used. We have considered the three dimensions of AEI (i.e., the perception of emotions, understanding of emotion and management of emotions) and have also added a fourth transversal dimension with examples of theoretical knowledge that represents a prerequisite to dAEI.

Some selected items of DC-knowledge (i.e., “understanding the difference between disinformation and misinformation”; “being aware of the meaning of non-verbal messages used in digital environments and knowing that their use can culturally differ between countries and communities”; Vuorikari et al., 2022) illustrate well how DC-Knowledge can have an impact on the perception of emotions. Knowing the meaning of non-verbal messages used in digital environments, and knowing the difference between disinformation and misinformation can consequently improve people's ability to perceive emotions online in several ways: by enabling them to better recognize the emotions of others through non-verbal cues, and by allowing them to better assess the intentions or motivations behind the information that they are receiving.

Some selected items of DC-knowledge (i.e., “knowing that AI systems can be used to automatically create digital using existing digital content as its source”; “knowing signs of digital addictions and that digital addiction can cause psychological and physical harm”; Vuorikari et al., 2022) illustrate well how DC-Knowledge can have an impact on the understanding of emotions. Knowing that AI systems can be used to automatically create digital content which may be difficult to distinguish from human creations can help people to better understand the potential emotional impacts of interacting with AI systems. It can consequently improve their understanding of their own emotions and enhance their sensitivity toward potential emotional impacts of these interactions. Knowing signs of digital addictions (e.g., loss of control, withdrawal symptoms, dysfunctional mood regulation) and their consequences can also improve people's ability to understand their own emotions when using (too much of) digital technologies. This can also be true regarding others: such knowledge may help one to be more aware of the emotions of others who may be experiencing digital addiction and in turn improve their understanding of emotions.

Several items of DC-knowledge may be relevant to explain management of emotions (i.e., “being aware that search engines, social media and content platforms often use AI algorithms to generate responses that are adapted to the individual user”; “being aware that adapting one's behavior in digital environments depends on one's relationship with other participants and the purpose in which the communication takes place”; Vuorikari et al., 2022). Knowing that search engines often use AI algorithms to generate responses adapted to the user can help them be more aware of the potential emotional impacts of interacting with these platforms and can consequently improve people's management of emotions. Knowing that one's behavior in digital environments should depend (1) on one's relationship with others and (2) on the specificities of said digital environments can help people better understand the ways in which emotions can be effectively managed in different social and professional contexts online and can consequently improve people's management of emotions.

Finally, some selected items of DC-knowledge may be considered as prerequisites to dAEI (i.e., “knowing the main functions of the most common digital devices; knowing some reasons why a digital device may fail to connect online”; “being aware that difficulties experienced while interacting with digital technologies may be due to technical issues, lack of confidence, one's own competence gap or inadequate choice of digital tool to solve the problem in question”; Vuorikari et al., 2022). Indeed, without such knowledge, there is no access to the digital world in the first place. Knowing the main functions of the most common digital devices and knowing some reasons why a digital device may fail to connect online is a prerequisite to dAEI as a minimum level of knowledge is needed to interact with digital technologies. Without this knowledge, it may be difficult or impossible for people to access and use digital technologies, and as such to develop dAEI. Knowing that difficulties experienced while interacting with digital technologies may be due to technical issues, lack of confidence or competence is also a prerequisite to dAEI as such knowledge is essential for being able to effectively troubleshoot and overcome challenges that may arise when using digital technologies. Without this understanding, people may be less able to access and use digital technologies, which can limit their participation in the digital world.

These examples provide an illustration of the way in which DC-Knowledge can play a role in enhancing and improving the different dimensions of dAEI. Altogether, this exercise shows how DC-Knowledge can help develop dAEI.

Some selected items of DC-Skills (i.e., “knowing how to analyze and critically evaluate search results and social media activity streams, to identify their origins, to distinguish fact-reporting from opinion, and to determine whether outputs are truthful or have other limitations”; “knowing how and when to use machine translation solutions and simultaneous interpretation apps to get a rough understanding of a document or conversation, but also knowing that when the content requires an accurate translation a more precise translation may be needed”; Vuorikari et al., 2022) illustrate well how DC-Skills can have an impact on the perception of emotions. Having the skills to analyze and critically evaluate search results and social media activity streams can help people to better understand the context and perspective behind different types of online information and content and can consequently improve people's perception of their own emotions as well as the perceptions of others' emotions. Knowing how and when to use machine translation solutions to get an understanding of a document or conversation can help people better understand the emotional content of such content and can consequently improve people's perception of emotions.

Our model further posits that DC-Skills may predict understanding of emotions. We believe this is particularly true for the following items (i.e., “knowing how to curate content on content sharing platforms so as to add value for oneself and others”; “knowing how to recognize embedded user experience techniques designed to manipulate and/or to weaken one's ability to be in control of decisions”; Vuorikari et al., 2022). Being able to curate content on content sharing platforms can allow people to better understand the interests and perspectives of the people they are interacting with and consequently improve their understanding of others' emotions. Being able to recognize embedded user experience techniques can allow people to better understand the motivations and intentions behind certain online interactions or behaviors and can consequently improve their understanding of others' emotions.

Some selected items of DC-Skills (i.e., “knowing how to adopt information and communication practices in order to build a positive online identity”; “being able to apply and follow protection strategies to fight online victimization”; Vuorikari et al., 2022) illustrate well how DC-Skills can have an impact on the management of emotions. Being able to adopt information and communication practices that build a positive online identity can allow people to present themselves in a way that reflects the emotions they want to display and can consequently improve the way they manage their own emotions. Being able to apply and follow protection strategies to fight online victimization can allow people to take control of their online interactions and protect themselves from harmful or negative experiences and can consequently improve the way they manage their emotions. Both skills can also help people show consideration for the wellbeing and safety of those they are interacting with online and thus improve the way they manage the emotions of others.

Finally, some selected items of DC-Skills (i.e., “knowing how to identify and solve a camera and/or a microphone issue when in an online meeting”; “taking a step-by-step approach to identify the root of a technical problem and exploring various solutions when facing a technical malfunction”; Vuorikari et al., 2022) illustrate well how DC-Skills can have a broader impact on dAEI and can be considered as prerequisites to dAEI in that, without them, there is no access to the digital world in the first place. The skills listed in the examples are essential for accessing the digital world and show the importance of developing DC-Skills through training. Being able to identify and solve problems with cameras and microphones, for example, is important for participating in online meetings and communication. Having the skills to take a step-by-step approach to problem-solving and explore various solutions can help individuals effectively troubleshoot technical issues. Being able to find solutions on the internet is also a valuable skill, as it allows individuals to access a wealth of information and resources that can help them resolve technical problems and access the digital world.

These examples provide an illustration of the way in which DC-Skills can play a role in enhancing and improving the different dimensions of dAEI. Altogether, this exercise shows how DC-Knowledge can help develop dAEI. In the following section we empirically test one of the conceptual links developed above, more specifically, how DC-Attitudes are predicted by TEI.

## 3. Empirical premises of digital emotional intelligence

### 3.1. Materials and methods

#### 3.1.1. Participants and procedure

Participants were recruited on Mturk and completed the questionnaires on Qualtrics. We required participants to (1) have a HIT (human intelligence task) approval rate of more than 95%, (2) be located US and (3) be currently employed. Among the whole sample, 40.9% of the participants were male and most of the participants between 25 and 45 years old (63.5%). Participants were first asked to provide their consent to participate in the study. Then, they reported information regarding their age, gender, employment status and flexible work possibilities. Participants were then presented with DigComp (Clifford et al., 2020) and TeiqueSF scales (Petrides and Furnham, 2001). Within each scale, we included five validity items randomly presented to control for participants' concentration during the survey. After removing participants who did not correctly answer validity items, our final sample consisted of 503 participants.

#### 3.1.2. Measures

Digital competence was measured using the DigComp framework (Clifford et al., 2020). This framework was selected for its global relevance, as opposed to van Laar et al. (2018) instrument that focuses on a specific category of professionals. Participants were asked to answer on a scale from 1 (“Strongly disagree”) to 5 (“Strongly agree”) to assess their perceived competence regarding digital competence. The questionnaire consists of 82 items measuring DC-Attitudes as well as DC-Knowledge and DC-Skills across the 5 subscales of DC (information, communication and collaboration, problem solving, safety, digital content creation). In this paper, we focus on the items measuring attitudes (21 items, alpha = 0.85). We thus had items measuring attitudes toward information (e.g., “I critically check if the information I find online is reliable”), communication and collaboration (e.g., “I am open toward sharing digital content that I think might be interesting and useful to others”), problem solving (e.g., “I am interested in understanding how a task can be broken down into steps so that it can be automated, for example in software or by a robot”), safety (e.g., “I am careful about checking the privacy policies of the digital services that I use”), and digital content creation (e.g., “I am interested in understanding how a task can be broken down into steps so that it can be automated, for example in software or by a robot”). We further aggregated the items for each subscale.

**Figure 2:**
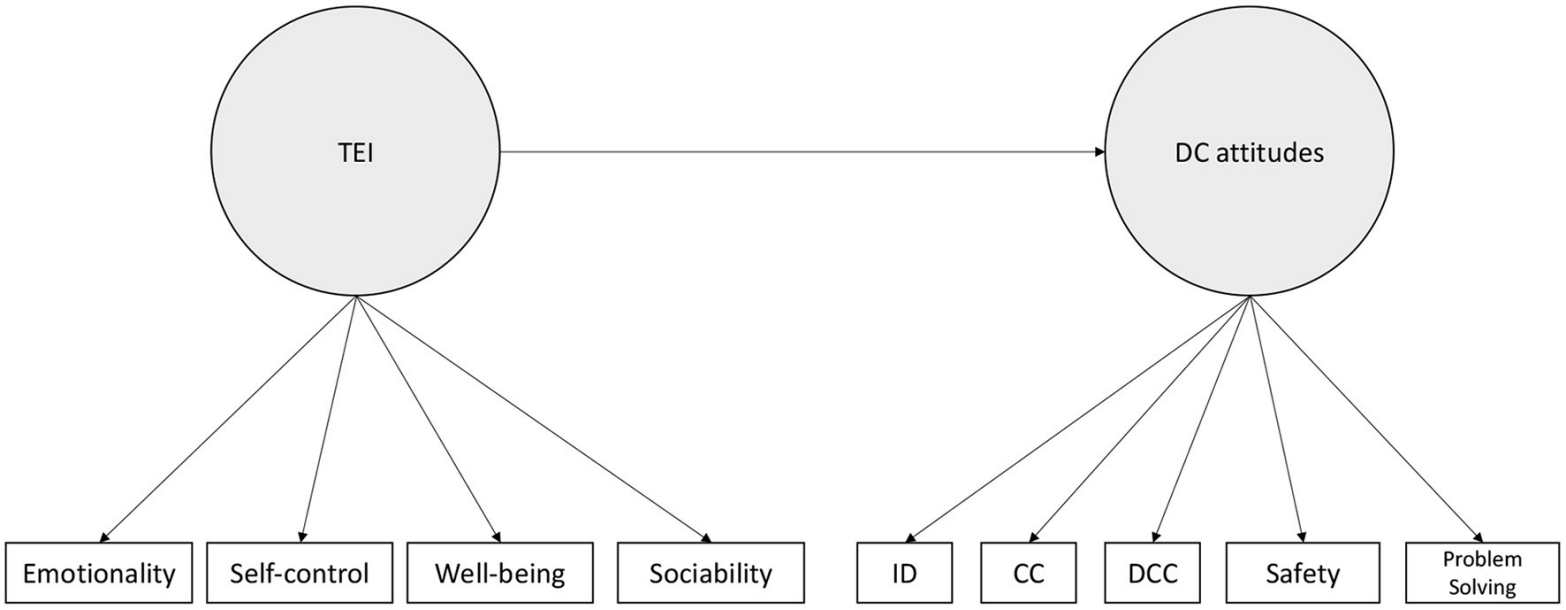
Hypothesized link between TEI and DC-attitudes. ID, Information and Data Literacy; CC, Communication and Collaboration; DCC, Digital Content Creation.

EI was measured using the TeiqueSF (Petrides and Furnham, 2001). This questionnaire distinguishes between four dimensions of EI: Emotionality (6 items such as “Expressing my emotions with words is not a problem for me” alpha = 0.84), Self-Control (6 items such as “I usually find it difficult to regulate my emotions”—reversed item I'm usually able to find ways to control my emotions when I want to”, alpha = 0.74), Wellbeing (6 items such as “I feel that I have a number of good qualities”, alpha = 0.62) and Sociability (6 items such as “I can deal effectively with people”, alpha = 0.74). The hypothesized model is depicted in Figure 2 below.

#### 3.1.3. Statistical analyses

Analyses were performed in R using *lavaan* (Rosseel et al., 2020). We first performed a two-factor confirmatory factor analysis (CFA) to test the existence of the two factors hypothesized in Figure 2 as well as their correlation (as in Sergi et al., 2007). In this model, we introduced TEI dimensions (emotionality, self-control, wellbeing and sociability) as well as the DC-Attitudes (information, communication and collaboration, problem solving, safety and digital content creation). We then performed a structural equation model (SEM) in which we introduced the same factors as in the CFA but we tested here the regression coefficient between TEI and DC-Attitudes. To assess the fit of the model, we used the comparative fit index (CFI), the standardized root square mean residual (SRMR), the root mean square error of approximation (RMSEA) and the Tucker-Lewis Index (TLI). We expected a good model to have a CFI above.95, an SRMR below.08, an RMSEA below.08 and a TLI above 0.90 (or above 0.95 to provide a good fit).

### 3.2. Results

We first report correlation matrix between the subdimensions of DC-Attitudes and with the dimensions of EI (i.e., wellbeing, sociability, emotionality, and self-control—see Table 1). Results highlight that all subdimensions of DC-Attitudes are positively related to each other (*r* = [0.416; 0.708]). This is also true for the dimension of EI (*r* = [0.609; 0.717]). Interestingly, the wellbeing dimension of EI is significantly and positively related to DC-attitudes (*r* = [0.288; 0.390]). Sociability is also related to most dimensions of DC-Attitudes (*r* = [0.162; 0.241]), except for the communication and collaboration attitudes (*r* = 0.086, *p* = 0.053). This is also true for the self-control dimensions, which is positively correlated with the DC-Attitudes (*r* = [0.09; 0.168], except for the communication and collaboration (*r* = −0.72, *p* = 0.613). Finally, emotionality is positively correlated with DC-Attitudes related to identification (*r* =.163, *p* < 0.001), safety (*r* = 0.095, *p* = 0.033), and problem-solving (*r* = 0.113, *p* = 0.011).

**Table 1:** Correlation coefficients between DC-Attitudes and TEI sub-dimensions.

	**Information and data literacy**		**Collaboration and communication**		**Digital content creation**		**Safety**		**Problem solving**		**Well-being**		**Sociability**		**Emotionality**		**Self-control**	
Information and data literacy	—																	
Communication and collaboration	0.467	***	—															
Digital content creation	0.416	***	0.652	***	—													
Safety	0.439	***	0.553	***	0.586	***	—											
Problem solving	0.512	***	0.645	***	0.708	***	0.627	***	—									
Wellbeing	0.288	***	0.227	***	0.324	***	0.353	***	0.390	***	—							
Sociability	0.162	***	0.086		0.224	***	0.193	***	0.241	***	0.649	***	—					
Emotionality	0.163	***	−0.072		0.077		0.095	*	0.113	*	0.622	***	0.613	***	—			
Self-control	0.168	***	−0.023		0.090	*	0.143	**	0.124	**	0.639	***	0.609	***	0.717	***	—	

^*^p < 0.05, ^**^p < 0.01, ^***^p < 0.001.

In the following, we report the results from the CFA model presented in Figure 2. Factors loadings are reported in Table 2 below. These results highlight that (1) all the hypothesized dimension load on their respective factors and (2) that TEI and DC-Attitudes are positively correlated (b = 0.26, 95%CI = [0.15; 0.32], z = 4.16, *p* < 0.001).

**Table 2:** Factor loadings for the two factors CFA linking DC-Attitudes and TEI.

**Factor loadings**	**Estimate**	**Standard Error**	**z-value**	***p*-value**	**95% ci (lower)**	**95% ci (upper)**	**Standardized estimate**
**DC-attitudes**							
Information and digital literacy	1.00	0.00			1.00	1.00	0.58
Communication and collaboration	0.97	0.09	11.4	0	0.80	1.14	0.77
Digital content creation	1.12	0.10	11.4	0	0.93	1.32	0.82
Safety	1.02	0.08	12.5	0	0.86	1.18	0.73
Problem solving	1.11	0.09	13.0	0	0.94	1.27	0.86

Emotionality	1.00	0.00			1.00	1.00	0.82
Wellbeing	0.87	0.05	18.3	0	0.78	0.96	0.80
Sociability	0.76	0.05	15.2	0	0.66	0.86	0.77
Self-control	0.84	0.05	17.7	0	0.75	0.94	0.82
**Correlation**							
DC-attitudes and TEI	0.11	0.03	4.2	0	0.06	0.16	0.26

**Figure 3:**
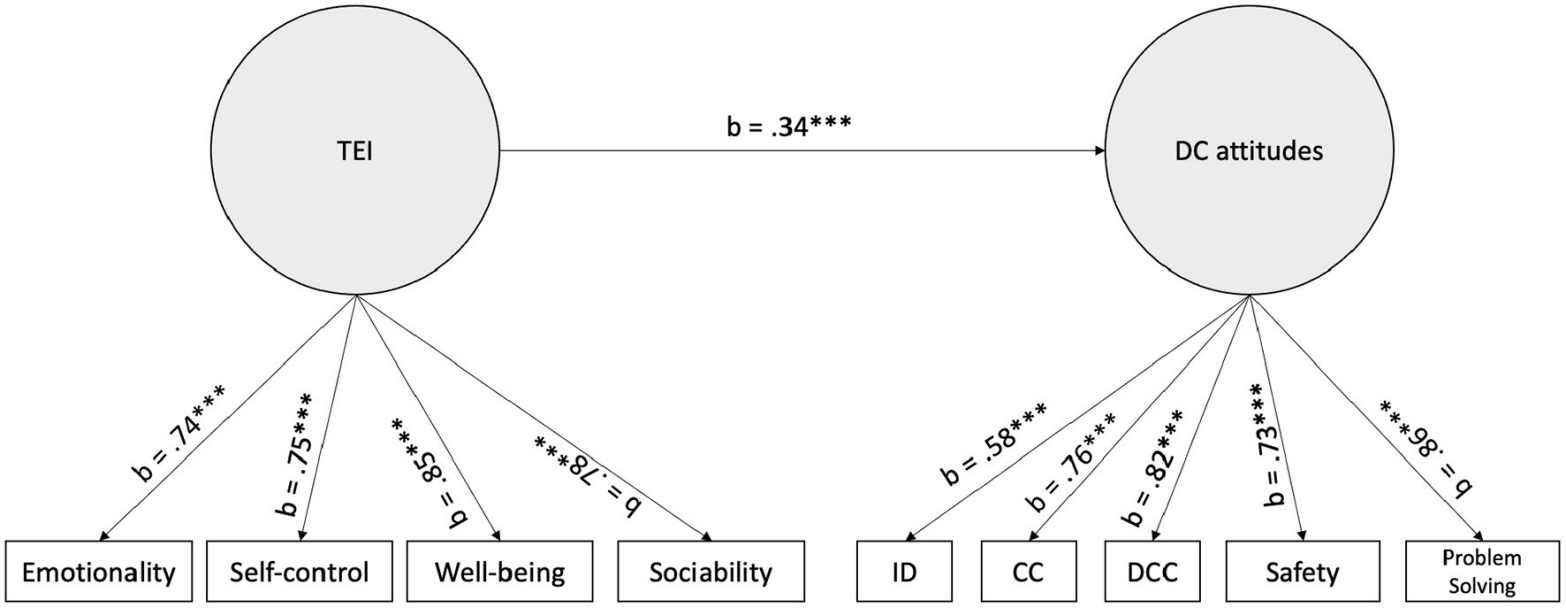
Tested links (standardized estimate) between TEI and DC-Attitudes. ID, Information and Data Literacy; CC, Communication and Collaboration; DCC, Digital Content Creation.

Below, we report the results from the SEM model, which is presented in Figure 3. Except for the RMSEA indices, the model provided an acceptable fit (RMSEA = 0.096, SRMR = 0.075, CFI = 0.954, TLI = 0.929). Factor loadings are reported in the Appendix. The results further highlight that trait EI significantly and positively predict DC-Attitudes (b = 0.339, 95%CI = [0.320; 0.340], z = 5.61, *p* < 0.001), suggesting that the higher trait EI, the more positive DC-Attitudes.

## 4. Discussion

The purpose of this work was to introduce the concept of “digital emotional intelligence” and propose it as a conceptual integration between EI and DC, emphasizing that it goes beyond solely EI in the digital context. Our conceptual model considers the two most prominent perspectives on EI (i.e., TEI and AEI). In terms of DC, we chose not to emphasize the different dimensions of DC (i.e., communication, digital content creation, safety or ethical issues) but instead focused on the transversal aspects of knowledge, skills and attitudes. Therefore, our model conceptualizes how EI and DC might impact each other to create dEI. We also conducted primary empirical testing of the relationship between EI and DC by focusing on how TEI can predict DC-Attitudes.

This work offers theoretical contributions to research on EI. Firstly, we build on the integrative conceptualization of EI, which combines both AEI and TEI (e.g., Mikolajczak, 2009). We believe that such a conceptualization provides a unifying view of EI. Although the term “digital emotional intelligence” already appears in the literature, the definitions used rather tend to rely on the sole ability model (Na-Nan et al., 2019). In this paper, we adopt a more global and unified view of dEI.

By conceptualizing dEI, we suggest that it is distinct from “traditional” EI and thus requires specific attention and measures. However, further testing is needed to ensure that “traditional” EI is indeed distinct from dEI. Additionally, the relationship between dEI and outcomes related to the digital world (in a professional or learning context) needs further investigation considering the new model of dEI. Lastly, we propose that the relationships between EI and DC are reciprocal, thereby contributing to research on EI by distinguishing between TEI as an antecedent and dAEI as an outcome of DC.

This paper offers a premise of empirical testing of the link between TEI and DC-Attitudes in DigComp. The primary results indicate a significant relationship between the two constructs, suggesting that TEI positively predicts DC-Attitudes. The relationship between DC-Knowledge and -Skills and dAEI requires further construct development and testing, as dAEI is proposed to be distinct from AEI.

This work makes a significant theoretical contribution to research on DC by highlighting its relationship with EI and emphasizing its relevance for dEI. Although there are some mentions of dEI in the literature (Oluwatofunmi and Amietsenwu, 2019; Sarnok et al., 2021), the embeddedness of the construct in a global digital competence framework has rarely been theoretically discussed. Traditional DC frameworks often do not explicitly consider emotions and EI. As shown previously, DigComp (Vuorikari et al., 2022) makes use of several examples that can be tied with a form of EI, but the link is not explicitly made. By elucidating this relationship, it becomes possible to investigate it and question how to study DC and EI jointly. More specifically, by suggesting distinct types of relationships between different forms of EI and different dimensions of DC, eventually with different frameworks, this paper offers a more comprehensive reflection on the various components of DC and their development and assessment.

This research also offers several practical implications. Almost 30 years ago, Goleman raised awareness of the importance of EI in the workplace (Goleman, 1995). In the intervening years, the world has undergone significant changes with the rise of mobile technologies, social media, and digital communication transforming the way we work, learn, and interact with others. As a result, it is crucial to update our understanding of EI to account for the specificities of the digital world and the knowledge and skills required to be “digitally emotionally intelligent.” This conceptual paper offers a renewed perspective on EI that aligns with contemporary notions of dEI.

This updated conceptualization has implications for policymakers, organizations, managers, and employees. Policymakers can integrate dEI more systematically into their frameworks and lead initiatives on DC that systematically account for dEI. Organizations can use this conceptualization to develop corporate training and policies that foster the development of dEI among their workforce. In particular, for remote work and virtual teams, raising awareness about the importance of dEI across the workforce is essential. Organizations can also consider dEI as a selection criterion for their workforce, especially for managerial positions. The conceptualization also highlights the importance of basic DC without which individuals cannot develop any form of dEI. It is therefore critical to foster the development of DC with adequate training initiatives at different levels (educational level, organizational level, etc.) to make sure that people are indeed able to develop dEI. The current conceptualization provides a spark of interest, but measurement instruments need to be developed to help institutions in their use of dEI. Organizations could thus improve their selection practices (for example to select remote workers who will require dEI in their day-to-day digital interactions at work). Measuring dEI would also be beneficial to organizations and training institutions in respect to digital learning as it would help them identify potential issues in their learners. This conceptualization can also help managers, employees, educators, and learners better understand the challenges they face in their work or learning environments Altogether, raising awareness of the concept of dEI makes it possible to consider DC and EI jointly into various programs and trainings to build a more digitally emotionally intelligent workforce and society.

This research offers several opportunities for future studies. Our model provides conceptual paths between TEI and DC-Attitudes and DC-Knowledge and DC-Skills and dAEI. Future studies are needed to (1) operationalize these concepts—specifically dAEI—and (2) test the relationships proposed in our model. By conceptualizing this new construct of dEI, this research also suggests that its relationship with various outcomes (organizational ones as well as learning ones) needs to be reassessed. For example, the relationship between collaborative behaviors, organizational citizenship behavior and dEI could be measured to account for specific digital learning setups and virtual teams. Another interesting line of research consists in the integration of a relatively new component of EI, namely emotion information processing (EIP; Fiori and Vesely-Maillefer, 2018). EIP refers to how people pay attention to, encode, retain, and retrieve information related to emotion (Vesely Maillefer et al., 2018). The specific articulation between EIP and dEI, and notably how EIP may or not be important in a digital context, and how it interacts with DC calls for future studies.

## 5. Conclusion

EI has been shown to be crucial in educational and organizational contexts as well as in everyday life. As more of our life takes place online, this paper proposes to formally coin “digital emotional intelligence” as an integration between EI and DC. The framework builds on both trait EI and ability EI and associates them with the knowledge, skills, and attitudes of DC. The paper also provides empirical premises on the relationship between TEI and DC-Attitudes, suggesting that TEI positively predicts DC-Attitudes, and highlights the importance of further investigating the relationship between EI and DC, especially regarding dAEI and DC-Knowledge and Skills. The framework proposed in this study provides a foundation for future research on dEI and its impact on work, learning, and everyday life. By pointing out the specificities of the digital context and the importance of DC in dEI, this paper develops the concept of dEI as more than just “emotional intelligence online.”

## Data availability statement

The raw data supporting the conclusions of this article will be made available by the author upon request.

## Ethics statement

Ethical review and approval was not required for the study on human participants in accordance with the local legislation and institutional requirements. The patients/participants provided their written informed consent to participate in this study.

## Author contributions

CA: study conception, data analysis, and manuscript writing. BA: study conception, data acquisition, and manuscript writing. All authors contributed to the article and approved the submitted version.

## Appendix

**Table:** Factor loadings for the SEM liking DC-attitudes and TEI.

**Factor loadings**	**Estimate**	**Standard Error**	**z-value**	***p*-value**	**95% ci (lower)**	**95% ci (upper)**	**Standardized estimate**
**DC-attitudes**							
Information and digital literacy	1.00	0.00			1.00	1.00	0.58
Communication and collaboration	0.97	0.08	11.4	0	0.80	1.13	0.77
Digital content creation	1.12	0.10	11.3	0	0.93	1.31	0.82
Safety	1.02	0.08	12.5	0	0.86	1.18	0.73
Problem solving	1.11	0.09	12.9	0	0.94	1.28	0.86
**TEI**							
Emotionality	1.00	0.00			1.00	1.00	0.74
Wellbeing	1.02	0.08	13.0	0	0.87	1.18	0.85
Sociability	0.86	0.07	12.8	0	0.73	0.99	0.79
Self-control	0.85	0.05	16.8	0	0.75	0.95	0.75
**Residual covariance**							
Wellbeing and self-control	0.14	0.05	3.0	0	0.05	0.23	0.37

## References

[B1] Aslan A. Zhu C. (2017). Investigating variables predicting Turkish pre-service teachers' integration of ICT into teaching practices. Br. J. Edu. Technol. 48, 552–570. 10.1111/bjet.12437

[B2] Audrin C. Audrin B. (2022). Key factors in digital literacy in learning and education: a systematic literature review using text mining. Edu. Inform. Technol. 3, 5. 10.1007/s10639-021-10832-5

[B3] Baartman L. K. De Bruijn E. (2011). Integrating knowledge, skills and attitudes: Conceptualising learning processes towards vocational competence. Edu. Res. Rev. 6, 125–134. 10.1016/j.edurev.2011.03.001

[B4] Blanch-Hartigan D. Andrzejewski S. A. Hill K. M. (2016). “Training people to be interpersonally accurate,” in The Social Psychology of Perceiving Others Accurately, eds J. A. Hall, M. Schmid Mast, and T. V. West (Cambridge University Press), (pp. 253–269). 10.1017/CBO9781316181959.012

[B5] Clifford I. Kluzer S. Troia S. Jakobsone M. Zandbergs U. (2020). DigCompSat. A Self-reflection Tool for the European Digital Framework for Citizens. Seville site: Joint Research Centre.

[B6] Department for Education. (2019). Essential digital skills framework. GOV.UK. Available online at: https://www.gov.uk/government/publications/essential-digital-skills-framework/essential-digital-skills-framework

[B7] Ferrari A. (2012). Digital Competence in Practice: An analysis of Frameworks. Luxembourg: Publications Office of the European Union.

[B8] Fiori M. Vesely-Maillefer A. K. (2018). “Emotional intelligence as an ability: theory, challenges, and new directions,” in Emotional Intelligence in Education: Integrating Research with Practice, eds K. V. Keefer, J. D. A. Parker, and D. H. Saklofske (Springer International Publishing), (pp. 23–47). 10.1007/978-3-319-90633-1_2

[B9] Garner P. W. (2010). Emotional competence and its influences on teaching and learning. Educ. Psychol. Rev. 22, 297–321. 10.1007/s10648-010-9129-4

[B10] Goleman D. (1995). Emotional Intelligence. Bantam Books, Inc. (pp. xiv, 352).

[B11] Hämäläinen R. Nissinen K. Mannonen J. Lämsä J. Leino K. Taajamo M.. (2021). Understanding teaching professionals' digital competence: what do PIAAC and TALIS reveal about technology-related skills, attitudes, and knowledge? Comput. Human Behav. 117, 106672. 10.1016/j.chb.2020.106672

[B12] Mayer J. D. Caruso D. R. Salovey P. (1999). Emotional intelligence meets traditional standards for an intelligence. Intelligence, 27, 267–298. 10.1016/S0160-2896(99)00016-112934682

[B13] Mayer J. D. Caruso D. R. Salovey P. (2016). The ability model of emotional intelligence: principles and updates. Emot. Rev. 8, 290–300. 10.1177/175407391663966724382883

[B14] Mayer J. D. Salovey P. (1997). “What is emotional intelligence?” in Emotional Development and Emotional Intelligence: Educational Implications. (Basic Books), (pp. 3–34).

[B15] Miao C. Humphrey R. H. Qian S. (2017a). A meta-analysis of emotional intelligence and work attitudes. J. Occup. Organ. Psychol. 90, 177–202. 10.1111/joop.12167

[B16] Miao C. Humphrey R. H. Qian S. (2017b). Are the emotionally intelligent good citizens or counterproductive? A meta-analysis of emotional intelligence and its relationships with organizational citizenship behavior and counterproductive work behavior. Pers. Individ. Dif. 116, 144–156. 10.1016/j.paid.2017.04.015

[B17] Mikolajczak M. (2009). Going beyond the ability-trait debate: the three-level model of emotional intelligence. E-J. Appl. Psychol. 5, 25–31. 10.7790/ejap.v5i2.175

[B18] Na-Nan K. Roopleam T. Wongsuwan N. (2019). Validation of a Digital Intelligence Quotient Questionnaire for Employee of Small and Medium-Sized Thai Enterprises Using Exploratory and Confirmatory Factor Analysis. Kybernetes. 10.1108/K-01-2019-0053

[B19] Oberländer M. Beinicke A. Bipp T. (2020). Digital competencies: a review of the literature and applications in the workplace. Computers and Education, 146, 103752. 10.1016/j.compedu.2019.103752

[B20] O'Connor P. J. Hill A. Kaya M. Martin B. (2019). the measurement of emotional intelligence: a critical review of the literature and recommendations for researchers and practitioners. Front. Psychol. 10, 1116. 10.3389/fpsyg.2019.01116 31191383 PMC6546921

[B21] Olderbak S. Semmler M. Doebler P. (2019). Four-branch model of ability emotional intelligence with fluid and crystallized intelligence: a meta-analysis of relations. Emotion Rev. 11, 166–183. 10.1177/1754073918776776

[B22] Oluwatofunmi A. D. Amietsenwu B. V. (2019). Relationship between digital emotional intelligence and performance of real estate digital marketing in Nigeria. Int. J. Psychol. Cog. Sci. 5, 70–78.

[B23] Petrides K. V. (2010). Trait emotional intelligence theory. Ind. Organ. Psychol. 3, 136–139. 10.1111/j.1754-9434.2010.01213.x

[B24] Petrides K. V. Furnham A. (2001). Trait emotional intelligence: psychometric investigation with reference to established trait taxonomies. Eur. J. Pers., 15, 425–448. 10.1002/per.416

[B25] Petrides K. V. Mikolajczak M. Mavroveli S. Sanchez-Ruiz M-. J. Furnham A. Pérez-González J-. C.. (2016). Developments in trait emotional intelligence research. Emotion Rev. 8, 335–341. 10.1177/1754073916650493

[B26] Rivers S. E. Brackett M. A. Salovey P. Mayer J. D. (2007). “Measuring emotional intelligence as a set of mental abilities,” in The Science of Emotional Intelligence: Knowns and Unknowns. (Oxford University Press) (pp. 230–257). 10.1093/acprof:oso/9780195181890.003.000936389024

[B27] Rosseel Y. Jorgensen T. D. Rockwood N. Oberski D. Byrnes J. Vanbrabant L.. (2020). lavaan: Latent Variable Analysis. 0, 6–7. Available online at: https://CRAN.R-project.org/package=lavaan

[B28] Salovey P. Mayer J. D. (1990). Emotional intelligence. Imagin. Cogn. Pers. 9, 185–211. 10.2190/DUGG-P24E-52WK-6CDG

[B29] Sánchez-Caballé A. Gisbert-Cervera M. Esteve-Mon F. (2020). (2020). The digital competence of university students: a systematic literature review. Aloma: Revista de Psicologia, Ciències de l'Educació i de l'Esport, 38, 63–74. 10.51698/aloma.2020.38.1.63-74

[B30] Sarnok K. Wannapiroon P. Nilsook P. (2021). Digital emotional intelligence (DEI) and learning achievement through digital storytelling in digital learning ecosystem for student teachers. 2021 5th International Conference on Education and Multimedia Technology 30–37. 10.1145/3481056.3481099

[B31] Sergi M. J. Rassovsky Y. Widmark C. Reist C. Erhart S. Braff D. L.. (2007). Social cognition in schizophrenia: relationships with neurocognition and negative symptoms. Schizophr. Res. 90, 316–324. 10.1016/j.schres.2006.09.028 17141477

[B32] Seufert S. Guggemos J. Sailer M. (2021). Technology-related knowledge, skills, and attitudes of pre-and in-service teachers: the current situation and emerging trends. Comput. Human Behav. 115, 106552. 10.1016/j.chb.2020.106552 32921901 PMC7474908

[B33] Sillat L. H. Tammets K. Laanpere M. (2021). Digital competence assessment methods in higher education: a systematic literature review. Edu. Sci. 11, 402. 10.3390/educsci11080402

[B34] Smith W. Wadley G. Webber S. Tag B. Kostakos V. Koval P.. (2022). Digital Emotion Regulation in Everyday Life. CHI Conference on Human Factors in Computing Systems 1–15. 10.1145/3491102.3517573

[B35] Szczygiel D. D. Mikolajczak M. (2018). Emotional intelligence buffers the effects of negative emotions on job burnout in nursing. Front. Psychol. 9, 2649. 10.3389/fpsyg.2018.02649 30627113 PMC6309155

[B36] Thatcher S. M. B. Fisher G. (2022). From the editors—The nuts and bolts of writing a theory paper: a practical guide to getting started. Acad. Manag. Rev. 47, 1–8. 10.5465/amr.2021.0483

[B37] van Laar E. van Deursen A. J. A. M. van Dijk J. A. G. M. Haan de. (2018). J. 21st-century digital skills instrument aimed at working professionals: conceptual development and empirical validation. Telem. Inform. 35, 2184–2200. 10.1016/j.tele.2018.08.006

[B38] Vesely Maillefer A. Udayar S. Fiori M. (2018). Enhancing the prediction of emotionally intelligent behavior: the PAT integrated framework involving trait EI, ability EI, and emotion information processing. Front. Psychol. 9, 1078. 10.3389/fpsyg.2018.01078 30013496 PMC6036374

[B39] Vuorikari R. Kluzer S. Punie Y. (2022). DigComp 2, 2. *The Digital Competence Framework for Citizens—With new examples of knowledge, skills and attitudes* (Joint Research Centre (Seville Site) No. JRC128415).

[B40] Wadley G. Smith W. Koval P. Gross J. J. (2020). Digital emotion regulation. Curr. Dir. Psychol. Sci. 29, 412–418. 10.1177/0963721420920592

[B41] Zeidner M. Roberts R. D. Matthews G. (2002). Can emotional intelligence be schooled? A critical review. Educ. Psychol. 37, 215–231. 10.1207/S15326985EP3704_2

[B42] Zhao Y. Pinto Llorente A. M. Sánchez Gómez M. C. (2021). Digital competence in higher education research: a systematic literature review. Comp. Edu. 168, 104212. 10.1016/j.compedu.2021.104212 36568577 PMC9759745

